# Draft Genome Sequence of the Fast-Growing Bacterium *Vibrio natriegens* Strain DSMZ 759

**DOI:** 10.1128/genomeA.00648-13

**Published:** 2013-08-22

**Authors:** Isabel Maida, Emanuele Bosi, Elena Perrin, Maria Cristiana Papaleo, Valerio Orlandini, Marco Fondi, Renato Fani, Juergen Wiegel, Giovanna Bianconi, Francesco Canganella

**Affiliations:** Laboratory of Microbial and Molecular Evolution, Department of Biology, University of Florence, Florence, Italya; Departments of Microbiology and of Biochemistry and Molecular Biology, University of Georgia, Athens, Georgia, USAb; Laboratory of Agricultural and Environmental Microbiology, Department for Innovation in Biological, Agrofood and Forest Systems, Viterbo, Italyc

## Abstract

*Vibrio natriegens* is a Gram-negative bacterium known for its extremely short doubling time. Here we present the annotated draft genome sequence of *Vibrio natriegens* strain DSMZ 759, with the aim of providing insights about its high growth rate.

## GENOME ANNOUNCEMENT

*Vibrio natriegens* was originally isolated from salt marsh muds of Sapelo Island, GA, and described by Payne et al. in 1961 (1). Initially, this organism was taxonomically placed into the genus *Pseudomonas* (*P. natriegens*), later redescribed as *Beneckea natriegens*, and finally recognized as a member of the genus *Vibrio* by Austin et al. (1978) (2). Eagon (1962) was the first to describe the unusually short generation time (9.8 min) of *V. natriegens* and the optimal conditions for its cultivation in batch: incubation at 37°C in brain heart infusion (BHI) with vigorous shaking (3). Nowadays, 9.8 min is still the shortest doubling time ever observed in biological investigations, and interesting speculations can be made about the limits of growth rates in prokaryotic microorganisms.

We have previously performed the continuous cultivation of *V. natriegens* in a small (50-ml) glass fermenter in order to examine the growth rates under optimized conditions such as temperature, nutrients, and air supply (4, 5). Moreover, physiological tests (optimal temperature, optimal NaCl percentage, and optimal medium evaluation) were carried out to find the best growth conditions. In either BHI medium or in mineral medium plus varied amounts of organic nutrients (peptone and glucose ranging between 0.01 and 1%) in batch cultures and under low dilution rates in continuous culture, *V. natriegens* exhibited high cell yields. The fastest growth with doubling times below 7 min was obtained only when the optical density (OD) of liquid cultures was very low (OD at 600 nm of between 0.05 and 0.1) and the cultures were under strong aeration. Controls suggest that the observed short doubling time could not be explained through wall growth effects. The reported results lead to speculations on what is theoretically the highest growth rate for a prokaryotic microorganism.

In this study, the genome of *Vibrio natriegens* strain DSMZ 759 was determined to provide new knowledge about fast-growing bacteria. The *Vibrio natriegens* draft genome sequence was obtained after Illumina HiSeq2000 sequencing. The 19,576,618 reads (101 bp long) were trimmed with SolexaQA (6) and the resulting reads (average length of about 62 bp) were assembled with the Abyss software version 1.3.2 (7). The *V. natriegens* genome consists of 5,200,362 bp long, distributed over 173 contigs (>500 bp; average length, 30,060 bp).

The draft genome was annotated by the Rapid Annotations using Subsystems Technology (RAST) server (8) using Glimmer3 as a gene caller (9), which predicted 4,788 coding sequences (CDS), including 3,542 with a putative functional annotation, 14 rRNA-encoding genes, and 71 tRNA-encoding genes.

Since fast cell growth requires a high rate of protein synthesis, the knowledge of rRNA operon copy numbers and their promoter sequences could provide useful insights about the mechanisms of such short generation time. Using a homology search approach, we predicted a total of 12 putative rRNA operons; this number of operons is similar to that experimentally found by Aiyar et al. in another strain of *V. natriegens* (10).

### Nucleotide sequence accession numbers. 

This whole-genome shotgun project has been deposited at DDBJ/EMBL/GenBank under the accession number ATWU00000000. The version described in this paper is version ATWU01000000.
